# Impact of navigation assistance on perioperative outcomes in UKA: A nationwide propensity score‐matched cohort study

**DOI:** 10.1002/jeo2.70693

**Published:** 2026-03-18

**Authors:** Yu Mori, Kunio Tarasawa, Ryuichi Kanabuchi, Hidetatsu Tanaka, Naoko Mori, Kiyohide Fushimi, Toshimi Aizawa, Kenji Fujimori

**Affiliations:** ^1^ Department of Orthopaedic Surgery Tohoku University Graduate School of Medicine Sendai Miyagi Japan; ^2^ Department of Health Administration and Policy Tohoku University Graduate School of Medicine Sendai Miyagi Japan; ^3^ Department of Radiology Akita University Graduate School of Medicine Akita Japan; ^4^ Department of Health Policy and Informatics Institute of Science Tokyo Tokyo Japan

**Keywords:** deep vein thrombosis, navigation‐assisted surgery, nationwide database, propensity score matching, unicompartmental knee arthroplasty

## Abstract

**Purpose:**

Unicompartmental knee arthroplasty (UKA) offers several clinical advantages compared to total knee arthroplasty, including faster recovery and reduced complication rates. Navigation‐assisted UKA has been introduced to improve prosthesis alignment; however, comprehensive evaluations of perioperative complications, particularly in Asian populations, remain scarce.

**Methods:**

A nationwide, retrospective cohort study was conducted using the Japanese Diagnosis Procedure Combination database from 2016 to 2023. A total of 30,724 patients who underwent UKA were included, of whom 8096 (26.3%) received navigation‐assisted surgery. After 1:1 propensity score matching for age, sex, body mass index (BMI), Charlson Comorbidity Index, anaesthesia type and bilateral procedures, 8056 patients were retained in each group. Perioperative complications, including deep vein thrombosis, pulmonary embolism, cerebrovascular events, surgical site infections and periprosthetic fractures, as well as length of hospital stay, were compared between groups. Given the large sample size in our analysis, the significance level was set at *p* < 0.001.

**Results:**

The incidence of deep vein thrombosis was significantly higher in the navigation‐assisted group compared to the conventional group (7.8% vs. 5.3%, *p* < 0.0001). Multivariate logistic regression identified navigation assistance (odds ratio [OR]: 1.52, 95% confidence interval [CI]: 1.34–1.72) and general anaesthesia (OR: 2.15, 95% CI: 1.74–2.67) as independent risk factors for deep vein thrombosis. In contrast, the navigation‐assisted group had a significantly shorter hospital stay (21.3 ± 11.4 vs. 23.1 ± 11.1 days, *p* < 0.0001). Other complications did not differ significantly between groups.

**Conclusion:**

Navigation‐assisted UKA was associated with a higher overall incidence of perioperative complications, largely driven by an increased risk of deep vein thrombosis, while length of hospital stay was shorter compared with conventional UKA. These findings suggest that although navigation assistance may facilitate earlier discharge, careful perioperative management—particularly with respect to thromboembolic risk—is warranted.

**Level of Evidence:**

Level III.

**Abbreviations**
CI: confidence interval
DPC: Diagnosis Procedure Combination
OA: osteoarthritis
PS: propensity score
SMD: standard mean difference
UKA: unicompartmental knee arthroplasty

## INTRODUCTION

Unicompartmental knee arthroplasty (UKA) has demonstrated clinical outcomes comparable to, and in some instances superior to, those of total knee arthroplasty, including favourable early postoperative outcomes such as lower complication and reoperation rates [4, 5, 6, 7, 11, 31]. UKA is an established treatment for patients with isolated medial or lateral compartment osteoarthritis (OA) and offers advantages such as faster recovery and preservation of knee function [8, 27, 30]. Appropriate patient selection remains essential, with current consensus indicating that the pattern and severity of compartmental disease—rather than age or body mass index (BMI) alone—are the primary determinants of UKA candidacy [8, 10, 26, 30].

Navigation‐assisted UKA has been reported to improve the accuracy of component positioning compared with conventional techniques [9, 12, 25, 32]; however, its impact on clinical outcomes and perioperative complications remains controversial [1, 32]. Some reports noted early postoperative pain reduction and a lower rate of complications or reinterventions with navigation‐assisted techniques, but many studies observed comparable clinical outcomes across methods over medium‐ and long‐term follow‐up [1, 32]. While the use of navigation assistance has been associated with prolonged operative and anaesthesia times [2], studies conducted in Western populations have reported no significant increase in the risk of perioperative complications [3]. However, large‐scale investigations focusing on Asian populations remain limited.

The Japanese Diagnosis Procedure Combination (DPC) database serves as a valuable resource for large‐scale cohort studies investigating perioperative complications in orthopaedic surgery [15, 16, 18, 19, 20, 21, 22, 29]. However, comprehensive evaluations comparing UKA performed with and without navigation assistance remain limited. This study aimed to address this gap by analysing the incidence of in‐hospital perioperative complications among patients who underwent UKA with or without navigation assistance, using a nationwide claims‐based database in Japan. To assess the risk of perioperative complications between conventional and navigation‐assisted UKA, we conducted a large‐scale, nationwide propensity score (PS)‐matched retrospective cohort study using the Japanese DPC database. The primary outcome of this study was the incidence of perioperative complications—including deep vein thrombosis, pulmonary embolism, cerebrovascular events, surgical site infections and periprosthetic fracture—compared between the two groups. The secondary outcome was length of hospital stay. We hypothesized that navigation‐assisted UKA would be associated with differences in perioperative complication profiles and length of hospital stay compared with conventional UKA.

## METHODS

### Study design

This study was conducted in accordance with the Declaration of Helsinki and was reviewed and approved by the institutional review board of our institution (approval number: 2024‐1‐1026), with a waiver of additional informed consent. The DPC database contains only de‐identified patient information, and no personally identifiable data were accessible to the investigators. The dataset was retrospectively extracted from the Japanese DPC database, which includes inpatient demographic information, diagnoses coded using the International Classification of Diseases, 10th Revision (ICD‐10), procedures, anaesthesia records and prescribed medications [17]. Patients who underwent UKA were identified based on procedure codes and primary diagnosis, and those undergoing total knee arthroplasty or revision surgery were excluded. This study was conducted as part of a nationwide survey involving DPC‐participating hospitals between April 2016 and March 2023. Throughout this period, approximately 1100 institutions consistently submitted medical records and consented to their inclusion in the present study. This study analysed patients who underwent UKA with or without navigation assistance at participating institutions using a nationwide administrative database in Japan.

The primary outcome of this study was the incidence of perioperative complications, including deep vein thrombosis, pulmonary embolism, cerebrovascular events, surgical site infections and periprosthetic fractures. The secondary outcome was length of hospital stay. The primary indications for UKA were determined based on the ICD‐10, with a particular focus on knee joint deformities caused by OA. OA was identified using ICD‐10 codes M170–M175 and M179. Patients who underwent UKA with or without navigation assistance were identified based on three criteria: (1) the main diagnosis, (2) the principal reason for hospitalization and (3) the condition requiring the highest level of medical resource utilization. Patients who underwent total knee arthroplasty or revision knee arthroplasty were excluded. Cases involving simultaneous bilateral procedures were included. The use of antithrombotic agents was also evaluated as a covariate in the analysis.

### PS matching

This study compared perioperative complications between patients who underwent UKA with and without navigation assistance. To reduce selection bias, 1:1 PS matching was employed. The analysis accounted for potential confounders, including age, sex, BMI, simultaneous bilateral procedures, Charlson Comorbidity Index and type of anaesthesia. Model discrimination was assessed using the C‐statistic. PS was estimated and used for nearest‐neighbour matching without replacement, applying a caliper width of 0.2 times the standard deviation of the estimated scores, in accordance with established methods [28]. The matching procedure resulted in a well‐balanced cohort of patients who underwent UKA with or without navigation assistance, thereby allowing for a valid and reliable comparative analysis between the two groups.

### Statistical analysis

Clinical data are expressed as mean ± standard deviation. Differences in clinical characteristics between the navigation‐assisted and conventional UKA groups were evaluated using the *χ* ^2^ test for categorical variables and Student's *t* test for continuous variables, both before and after PS matching. Comparative analyses of perioperative complication risks between the two groups were similarly conducted using the *χ* ^2^ test and Student's *t* test in both the unmatched and matched cohorts.

Multivariate logistic regression analysis was performed to identify independent predictors of severe perioperative complications, adjusting for variables beyond the use of navigation assistance. This analysis revealed factors independently associated with adverse outcomes. Given the large sample size, a more stringent significance threshold was adopted. All statistical tests were two‐sided, with a *p* value of <0.001 considered statistically significant. Statistical analyses were conducted using JMP version 17 (SAS Institute).

## RESULTS

**Figure 1:**
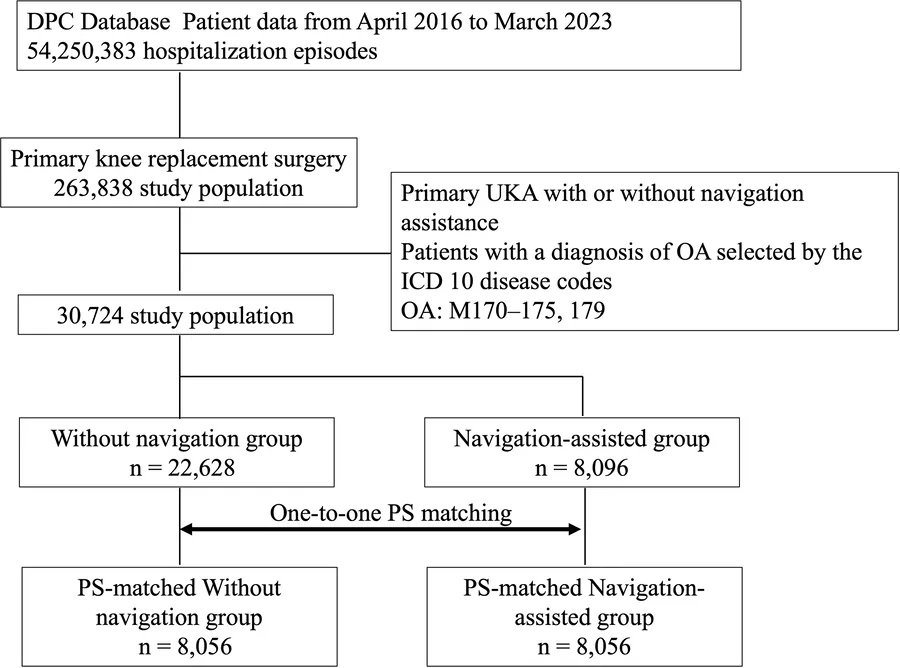
Flow diagram of patient selection and propensity score (PS) matching. Patients who underwent unicompartmental knee arthroplasty (UKA) with or without navigation assistance for osteoarthritis (OA) were identified from the Diagnosis Procedure Combination (DPC) database between April 2016 and March 2023. The diagram illustrates the patient selection process and the subsequent 1:1 PS matching. ICD 10, International Classification of Diseases, 10th Revision.

Figure 1 presents a schematic representation of the patient selection process. From the dataset covering the period from April 2016 to March 2023, a total of 30,724 patients who met the predefined inclusion and exclusion criteria were identified. Among them, 22,628 patients were categorized into the conventional UKA group, while 8096 (26.3%) were classified into the navigation‐assisted UKA group. Following PS matching based on variables such as age, sex, BMI, Charlson Comorbidity Index, presence of simultaneous bilateral procedures and type of anaesthesia, 8056 patients were retained in each group for comparative analysis.

Table 1 summarizes the baseline characteristics of patients who underwent UKA with or without navigation assistance. Prior to PS matching, notable imbalances were observed between the two groups, particularly in the type of anaesthesia, with a standardized mean difference (SMD) exceeding 0.1. The conventional UKA group had a higher proportion of patients receiving general anaesthesia. After 1:1 PS matching, all covariates achieved SMDs below 0.1, indicating satisfactory balance between the groups. The C‐statistic of the PS model was 0.898, indicating excellent discrimination between patients who underwent navigation‐assisted and conventional UKA.

**Table 1:** Characteristics of patients before and after PS matching.

	Before PS matching			After PS matching		
Confounding factors	Without navigation	Navigation‐assisted	SMD	Without navigation	Navigation‐assisted	SMD
*n*	22628	8096		8056	8056	
Sex						
Male	6179 (27.3%)	2308 (28.5%)	0.027	2354 (29.2%)	2302 (28.6%)	0.015
Female	16449 (72.7%)	5788 (71.5%)		5702 (70.8%)	5754 (71.4%)	
Age	74.4 ± 7.6	74.2 ± 7.6	0.027	74.3 ± 7.7	74.2 ± 7.6	0.012
Body mass index	25.3 ± 4.5	25.1 ± 3.6	0.047	25.2 ± 3.6	25.1 ± 3.6	0.008
Charlson comorbidity index	0.55 ± 0.86	0.50 ± 0.81	0.065	0.51 ± 0.82	0.50 ± 0.81	0.012
Bilateral surgery	869 (3.8%)	406 (5.0%)	0.057	392 (4.9%)	405 (5.0%)	0.007
General anaesthesia	20242 (89.5%)	6736 (83.2%)	0.184	6690 (83.0%)	6712 (83.2%)	0.007

*Note*: One‐to‐one PS matching was performed. Data are shown as mean ± standard deviation.

Abbreviations: PS, propensity score; SMD, standard mean difference.

Table 2 presents the use of anticoagulant and antiplatelet agents for thromboprophylaxis. Edoxaban was the most frequently prescribed agent, accounting for over 50% of prescriptions in both groups. Antithrombotic therapies were also administered based on clinical indications related to comorbid conditions. Aspirin use was notably higher in the navigation‐assisted UKA group. Aside from aspirin, no statistically significant differences were observed in the use of pharmacologic agents for the prevention of deep vein thrombosis and pulmonary embolism between the conventional and navigation‐assisted UKA groups, both before and after PS matching. Over 80% of patients in both groups received pharmacologic thromboprophylaxis, with edoxaban being the most commonly prescribed agent.

**Table 2:** Comparison of antithrombotic therapies before and after PS matching.

	Before PS matching			After PS matching		
	Without navigation	Navigation‐assisted	SMD	Without navigation	Navigation‐assisted	SMD
Edoxaban	11687 (51.7%)	4246 (52.5%)	0.016	4238 (52.6%)	4230 (52.5%)	0.002
Fondaparinux	610 (2.7%)	250 (3.1%)	0.023	216 (2.7%)	250 (3.1%)	0.025
Enoxaparin	1813 (8.0%)	656 (8.1%)	0.003	637 (7.9%)	655 (8.1%)	0.008
Aspirin	1857 (8.2%)	1265 (15.6%)	0.233	660 (8.2%)	1247 (15.5%)	0.229
Warfarin	273 (1.2%)	69 (0.9%)	0.035	98 (1.2%)	69 (0.9%)	0.036
Clopidogrel	619 (2.7%)	219 (2.7%)	0.002	209 (2.6%)	218 (2.7%)	0.007
Apixaban	382 (1.7%)	144 (1.8%)	0.007	129 (1.6%)	143 (1.8%)	0.014

*Note*: One‐to‐one PS matching was performed.

Abbreviations: PS, propensity score; SMD, standard mean difference.

Table 3 summarizes perioperative complication events before and after PS matching. Before matching, the total number of perioperative complication events was higher in the navigation‐assisted UKA group than in the conventional UKA group (715 [8.8%] vs. 1561 [6.9%], *p* < 0.0001). After PS matching, a similar pattern was observed, with a higher number of perioperative complication events in the navigation‐assisted group compared with the conventional group (714 [8.9%] vs. 529 [6.6%], *p* < 0.0001). These values represent the total number of complication events, and some patients may have experienced more than one event during hospitalization.

**Table 3:** Comparison of complications before and after PS matching.

	Before PS matching			After PS matching		
	Without navigation	Navigation‐assisted	*p* Value	Without navigation	Navigation‐assisted	*p* Value
Total perioperative complications	1561 (6.9%)	715 (8.8%)	<0.0001	529 (6.6%)	714 (8.9%)	<0.0001
Deep vein thrombosis	1255 (5.6%)	627 (7.7%)	<0.0001	424 (5.3%)	626 (7.8%)	<0.0001
Pulmonary embolism	26 (0.1%)	19 (0.2%)	0.016	10 (0.1%)	19 (0.2%)	0.09
Cerebrovascular disorder	59 (0.3%)	15 (0.2%)	0.23	17 (0.2%)	15 (0.2%)	0.72
Surgical site infection	184 (0.8%)	44 (0.5%)	0.015	59 (0.7%)	44 (0.6%)	0.14
Periprosthetic fracture	37 (0.2%)	10 (0.1%)	0.43	19 (0.2%)	10 (0.1%)	0.09
Length of hospitalization (days)	23.5 ± 11.4	21.3 ± 11.4	<0.0001	23.1 ± 11.1	21.3 ± 11.4	<0.0001

*Note*: One‐to‐one PS matching was performed. Total perioperative complication events represent the sum of individual complication events.

Abbreviation: PS, propensity score.

*p values of <0.001 are considered significant by the *χ* ^2^ test and Student's *t* test.

Deep vein thrombosis was the most frequent complication and occurred significantly more often in the navigation‐assisted group both before and after PS matching (after matching: 7.8% vs. 5.3%, *p* < 0.0001). In contrast, the length of hospital stay was shorter in the navigation‐assisted group both before and after matching; after matching, the mean length of stay was 21.3 ± 11.4 days in the navigation‐assisted group and 23.1 ± 11.1 days in the conventional group. The incidence of periprosthetic fracture was low in both groups (0.1% vs. 0.2%) and did not differ significantly between groups.

Table 4 presents the results of a multivariate logistic regression analysis investigating factors associated with the development of deep vein thrombosis following UKA with or without navigation assistance. Among the variables evaluated, both navigation assistance and the use of general anaesthesia emerged as significant independent risk factors for deep vein thrombosis. Specifically, navigation assistance was associated with an odds ratio (OR) of 1.52 (95% confidence interval [CI]: 1.34–1.72, *p* < 0.0001), while general anaesthesia was associated with an OR of 2.15 (95% CI: 1.74–2.67, *p* < 0.0001).

**Table 4:** Multivariate logistic analysis for risk factors for deep vein thrombosis after unilateral knee arthroplasty during hospitalization.

	Deep vein thrombosis	
Variable	Odds ratio (95% CI)	*p* Value
Age	1.00 (0.99–1.01)	0.79
Sex (female)	1.12 (0.97–1.29)	0.12
Body mass index	1.00 (0.98–1.02)	0.88
Bilateral surgery	1.35 (0.98–1.85)	0.064
General anaesthesia	2.15 (1.74–2.67)	<0.0001
Charlson comorbidity index	0.91 (0.84–0.99)	0.027
Navigation assistance	1.52 (1.34–1.72)	<0.0001

Abbreviation: CI, confidence interval.

*p values < 0.001 are considered statistically significant.

## DISCUSSION

In this nationwide PS‐matched cohort study, navigation‐assisted UKA was associated with a higher number of perioperative complication events compared with conventional UKA, while length of hospital stay was shorter in the navigation‐assisted group. These findings partially support our study hypothesis, indicating differences in perioperative complication profiles and postoperative hospital stay between the two surgical approaches. Regarding the primary outcome, the overall number of perioperative complication events was greater in the navigation‐assisted group, largely driven by an increased incidence of deep vein thrombosis. Navigation assistance was identified as an independent risk factor for deep vein thrombosis, whereas other individual complications occurred at similarly low rates between groups. In contrast, as a secondary outcome, the length of hospital stay was consistently shorter in patients undergoing navigation‐assisted UKA.

This large‐scale, nationwide study evaluated perioperative outcomes associated with navigation‐assisted versus conventional UKA using data from 30,724 procedures performed in Japan between 2016 and 2023. Among these, 8096 cases (26.3%) involved navigation assistance. More than 80% of patients in both the navigation‐assisted and conventional UKA groups received pharmacologic thromboprophylaxis, with edoxaban being the most frequently administered agent. After 1:1 PS matching based on key demographic and clinical variables—including age, sex, BMI, Charlson Comorbidity Index, type of anaesthesia and presence of simultaneous bilateral procedures—8056 patients were included in each group for balanced comparative analysis.

Two principal findings emerged. First, the incidence of deep vein thrombosis was significantly higher in the navigation‐assisted group than in the conventional group (7.8% vs. 5.3%, *p* < 0.0001), a difference that remained statistically significant after adjustment. Second, the navigation‐assisted group demonstrated a shorter hospital stay (21.3 ± 11.4 days) compared to the conventional group (23.1 ± 11.1 days, *p* < 0.0001).

Few nationwide studies have evaluated perioperative outcomes of navigation‐assisted UKA. Previous studies conducted predominantly in Western populations have focused primarily on implant alignment, functional outcomes and reoperation rates [1, 9, 12, 25, 32], while relatively few have systematically assessed perioperative complications [3, 32]. Moreover, those who did report complication outcomes often lacked robust adjustment for confounding factors. In contrast, the present study employed rigorous PS matching and multivariate regression, providing a more reliable estimate of the complication risk profile associated with navigation assistance.

The increased incidence of deep vein thrombosis in the navigation‐assisted group may be partly attributable to procedural factors. Navigation‐assisted UKA has been associated with prolonged operative and anaesthesia times [2], both of which are recognized risk factors for venous thromboembolism. While operative time was not available in the current dataset, our multivariate logistic regression analysis identified navigation assistance (OR: 1.52; 95% CI: 1.34–1.72) and general anaesthesia (OR: 2.15; 95% CI: 1.74–2.67) as independent risk factors for deep vein thrombosis. These findings emphasize the need for tailored perioperative management strategies in patients undergoing navigation‐assisted procedures. An additional consideration is the difference in antithrombotic regimens between groups. The navigation‐assisted group more frequently received aspirin, which may have influenced thromboembolic outcomes. Although aspirin is commonly used for venous thromboembolism prophylaxis after knee arthroplasty, differences in agent selection and dosing could not be fully accounted for in this claims‐based analysis.

On the other hand, the reduced hospital stay observed in the navigation‐assisted group may reflect benefits related to improved implant positioning, more precise soft tissue handling or enhanced early mobilization—advantages previously reported in studies examining radiographic and early clinical outcomes of navigation‐assisted UKA [1, 12, 25]. The relatively long length of hospital stay reflects the characteristics of the Japanese acute care system under the DPC framework, where inpatient rehabilitation and discharge planning are commonly included. Because both groups were treated within the same healthcare system, the between‐group difference in length of stay remains clinically meaningful. Although functional and patient‐reported outcome measures were not available in this claims‐based analysis, shorter hospitalization may indirectly suggest more favourable short‐term recovery.

Importantly, other serious complications—including pulmonary embolism, cerebrovascular events, surgical site infections and periprosthetic fractures—occurred at similarly low rates across both groups, underscoring the general safety of UKA regardless of navigation use when appropriate patient selection is applied.

A major strength of this study lies in the use of a nationwide administrative database in combination with PS matching, which allowed for effective adjustment for key confounding variables such as age, sex, BMI, type of anaesthesia, simultaneous bilateral procedures and comorbidities. In addition, the large sample size enhances the robustness and generalizability of the findings. Nonetheless, this study has several limitations. First, the study population was limited to patients who underwent UKA in acute care hospitals participating in the DPC data system. As a result, individuals treated in non‐DPC‐reported beds—representing approximately 30% of general hospital beds in Japan—as well as those who were never admitted to acute care hospitals, were not included [29]. Second, deep vein thrombosis diagnoses were based on administrative claims data, which may be subject to detection or coding bias, particularly if surveillance intensity differed between groups. Third, the accuracy of disease classifications within the DPC system and the severity of comorbid conditions could not be verified at the individual patient level. Fourth, hospital‐level factors such as institutional volume, level of care and surgeon experience were not available in the DPC database and therefore could not be included in the PS matching. Fifth, information on surgical duration and the use of a tourniquet was not available, despite both being reported as risk factors for deep vein thrombosis, pulmonary embolism and surgical site infection [13, 14, 23, 24]. Lastly, long‐term postoperative outcomes such as infection, periprosthetic fracture, reoperation and mortality after discharge were not assessed. Future large‐scale studies incorporating more granular clinical and surgical data, along with long‐term follow‐up, are needed to address these limitations and provide a more comprehensive understanding of postoperative risks.

## CONCLUSION

This nationwide PS‐matched cohort study demonstrated that navigation‐assisted UKA was associated with a higher overall incidence of perioperative complications, primarily attributable to an increased risk of deep vein thrombosis, while length of hospital stay was shorter compared with conventional UKA. While navigation‐assisted UKA may contribute to earlier discharge, heightened vigilance regarding perioperative thromboembolic complications is necessary.

## AUTHOR CONTRIBUTIONS

Yu Mori and Hidetatsu Tanaka conceived and designed the study. Yu Mori performed the statistical analyses and drafted the manuscript. Kunio Tarasawa, Ryuichi Kanabuchi and Naoko Mori contributed to data interpretation. Kiyohide Fushimi and Kenji Fujimori contributed to data acquisition and database management. Toshimi Aizawa supervised the study. All authors critically revised the manuscript and approved the final version.

## CONFLICT OF INTEREST STATEMENT

The authors declare no conflict of interest.

## ETHICS STATEMENT

This study was conducted in accordance with the Declaration of Helsinki and was approved by the Institutional Review Board of Tohoku University (approval number: 2024‐1‐1026).

## Data Availability

The data that support the findings of this study are available from the Japanese Diagnosis Procedure Combination (DPC) database. Restrictions apply to the availability of these data, which were used under license for the current study, and so are not publicly available.
